# Estimations on Properties of Redox Reactions to Electrical Energy and Storage Device of Thermoelectric Pipe (TEP) Using Polymeric Nanofluids

**DOI:** 10.3390/polym13111812

**Published:** 2021-05-31

**Authors:** Qin Gang, Rong-Tsu Wang, Jung-Chang Wang

**Affiliations:** 1 College of Chemistry and Chemical Engineering, Shanghai University of Engineering Science, Shanghai 201620, China; qin1862199@163.com; 2 Department of Leisure Management, Yu Da University of Science and Technology, Miaoli County 36143, Taiwan; 3 Department of Marine Engineering (DME), National Taiwan Ocean University (NTOU), Keelung 202301, Taiwan

**Keywords:** polymeric nanofluid, two-step synthesis, electrochemistry, redox reaction, thermal performance, thermoelectric pipe

## Abstract

A thermoelectric pipe (TEP) is constructed by tubular graphite electrodes, Teflon material, and stainless-steel tube containing polymeric nanofluids as electrolytes in this study. Heat dissipation and power generation (generating capacity) are both fulfilled with temperature difference via the thermal-electrochemistry and redox reaction effects of polymeric nanofluids. The notion of TEP is to recover the dissipative heat from the heat capacity generated by the relevant machine systems. The thermal conductivity and power density empirical formulas of the novel TEP were derived through the intelligent dimensional analysis with thermoelectric experiments and evaluated at temperatures between 25 and 100 °C and vacuum pressures between 400 and 760 torr. The results revealed that the polymeric nanofluids composed of titanium dioxide (TiO_2_) nanoparticles with 0.2 wt.% sodium hydroxide (NaOH) of the novel TEP have the best thermoelectric performance among these electrolytes, including TiO_2_ nanofluid, TiO_2_ nanofluid with 0.2 wt.% NaOH, deionized water, and seawater. Furthermore, the thermal conductivity and power density of the novel TEP are 203.1 W/(m·K) and 21.16 W/m^3^, respectively.

## 1. Introduction

High heat flux generated by many electronic products has recently been comprehensively causing hotspot problems that need to be improved immediately with novel technologies with excellent performances. At present, many 3-C (Computers, Consumers, and Communications) electronic products employ heat pipe [1,2,3,4,5] and vapor chamber [6,7,8,9,10,11] thermal modules to become the standard equipment, so as to increase the heat dissipation efficiency of the goods and reduce the temperature of the heat sources [12,13,14]. These two-phase flow heat transfer facilities have high thermal conductivities compared to those of the large-footprint metal material heat sinks [15]. Wang et al. [16,17] showed that the maximum three-dimensional and effective thermal conductivity of the vapor chamber is up to 910 W/mK, many times that of the pure copper base plate at heat flux of over 100 W/cm^2^. If it can lower the temperature of the heat source simultaneously and then recycle the heat dissipated, it will be a great contribution to the green energy industry on the basis of conserving energy. Nowadays, as shown in the present paper, it is possible to utilize the temperature effects on electrochemical and thermal activities [18] to accomplish this intention, which can supply a cooling function for the electronic devices and simultaneously exploit the wasted heat energy to generate direct current electric power. Tan et al. [19] found reasonable charging rates for lithium-ion battery management systems’ design at different initial temperatures employing the thermal-electrochemistry coupled model, which investigates the temperature effects on electrochemical and thermal characteristics.

The first voltaic cell/pile of the world was developed by Alessandro Volta, an Italian physicist, between the end of the 18th century and the beginning of the 19th century. It can gather and provide a stable current via the electrochemistry reduction-oxidation (redox) reaction. Hereafter, in 1832, Michael Faraday, an English scientist, depicted in detail the electrochemistry phenomena about the electrolysis process between electric energy and chemical decomposition through solution containing ions. Wang et al. [20,21,22] utilized the metal oxide nanofluids as an electrolyte (thermoelectric nanofluids) compared with various aqueous solutions according to pH value, Zeta potential, viscosity, and IEP to display the best particle fraction, stability, and settled current output. These results indicated that the thermoelectric nanofluids (Al_2_O_3_ nanofluid) with temperature variation make use of the temperature effects on electrochemical and thermal activities, which depict the thermoelectric conversion function of temperature gradient into electric power generation in order to improve the application rate of the wasted thermal energy. Nanofluids are widely considered as the innovative nanotechnology-based heat transfer fluids, which have been certified for reforming the energy conversion procedure efficiency [23,24]. The creative notion of thermoelectric nanofluids is energetic and extremely dependable energy transformation that generates electricity in applications in which the heat will be dissipated. For the recent developments in thermal and electrical conductivities of this novel thermal fluid, various factors affect it, including the cluster of nanoparticle type, temperature, preparation methods, surfactant, and volume concentration. It was found that the rise in temperature and volume concentration of nanofluids generally led to linearly incrementing their thermal and electrical conductivities [25,26,27]. Heyhat and Irannezhad [28] created the thermoelectrical conductivity (TEC) ratio according to the acquired experimental data and thoroughly checked it. Results displayed that both the temperature and concentration have affirmative effects on the thermal and electrical conductivities of nanofluids. Geng et al. [29] discussed the effects of base fluid, temperature, solid volume concentration, and nanoparticle type on electrical conductivity of nanofluid and found that the electrical conductivity generally increases as temperature and solid volume fraction increase. Kim and Park [30] investigated the influence of the multi-walled carbon nanotubes (MWCNTs) nanofluid as an electrolyte on the energy storage capacity in vanadium redox flow battery, which was inspected and contrasted with the primitive electrolyte.

This study develops a thermoelectric pipe (TEP) device for the first time depending on the temperature effects on electrochemical and thermal activities having heat conduction performance and suitability, which adopted the polymeric nanofluid as an electrolyte. According to [21], the 2.0 wt.% titanium dioxide (TiO_2_) nanofluid had the best suspension stability and overall thermoelectric properties among the three nanofluids, including the Al_2_O_3_, ZnO, and TiO_2_, in the thermoelectric generation experiments. Pinchuk and Kuzmin [31] studied the effect of the addition of TiO_2_ nanoparticles to coal-water fuel on its thermophysical parameters. They suggested that the addition of the TiO_2_ nanoparticles in 0.5 to 4 wt.% increases the coal-water fuel thermal conductivity by 9% to 17%. Das et al. [32] noticed that use of TiO_2_ nanofluid reduces the wall temperature distribution as well as thermal resistance of thermosyphon and enhances the thermal conductivity compared to deionized water. Therefore, the nanofluids revealed higher thermal conductivity contrasted with base fluid and demonstrated an increase in the effective thermal conductivity with a reduction in particle size and with a growth in particle volume fraction. The present TEP composed of the polymeric nanofluid is capable of generating electromotive force and let-bearing heat dissipation at a temperature difference, and vice versa. Consequently, it is especially favorable for applications in the industrial waste heat and automotive waste heat used for recycling and reusing in order to reduce carbon dioxide emissions. The primary object of the present TEP is to provide a novel device that have high heat conduction performance and relatively good power generation suitability.

## 2. Materials and Methodology

The section interprets the structural design, thermoelectric function testing, and empirical formula derivation of the thermoelectric performance of the novel TEP, which working principle uses the concepts of heat conduction of the heat pipe and power generation of the electrochemical cell and employs the polymeric nanofluids as electrolysis liquid to improve the thermoelectric performance. Separately, deducing the thermal conductivity of the novel TEP by thermal resistance analysis and measuring the output current and power density of the novel TEP at different temperatures are exploited to derive the empirical formula of the thermoelectric performance via the intelligent dimensional analysis [22] with various experimental data.

### 2.1. Empirical Formula Derivation of the Novel TEP Thermoelectric Performance

Ordinarily, most of the engineering related to the heat-flow physical mechanics can be analyzed via the motion equations and underlying theories, but there are still many deductions that should be experimentally inspected in order to acquire realistic findings, because the deductions derived from the fundamental theories and motion equations may only be employed for the basic estimations. Units and scales are a manual conception with underlying relevance in the physical world, in which it is a more official way of signifying that kind thought. Accordingly, the dimensional analysis does not frequently render a whole exploration, yet it supplies the beneficial procedures toward an intact comprehension for exploiting the helpful results that are not petty and not distinct. Dimensional analysis is a variable skill and manner that may be accustomed to clarifying and demonstrating the conjunctions between physical quantities, and makes it possible to gather the consequences of estimations and tests in a concise and widespread formula, which can apply forecasts expeditiously. Dimensional analysis is actually adopted to assemble the consequences of experiments in simple and clear modus, so that we can achieve the ordinary fitting from a little number of examinations at a model scale. The present study obtains the empirical formulas of the novel TEP thermoelectric performance, which are derived from the experimental data and the dimensional analysis of factors in Vashy-Buckingham Pi (Π) Theorem. The dimensional analysis with intelligent experiment [22] was introduced, and the application of the empirical formula was searched for the electric charge density output of the novel TEP and waste heat development in the present work. The major ideology of the dimensional analysis with intelligent experiment is that the relationship can continuously be expressed as conjunctions between these Π-dimensional groups. The present study aims to illustrate that the dimensional analysis is a formidable means and covers extended, evident, and new achievements. The analysis procedure is performed to find out all the variables of the novel TEP thermoelectric performance through the repeating variable method resulting from the basic dimensional qualifications. For effective thermal conductivity and power generation empirical formulas of the novel TEP, the dimensional analysis procedure [22] was as follows:

(1) $$K_{tp}=Function\left\{K_{nf},C_{nf},F_{tp},\;T_{tp},P_{tp},\mu_{nf},\;\rho_{nf}\right\}$$

(2) $${\bar{P}}_{tp}=Function\left\{V_{n},K_{nf},F_{tp},\;\rho_{nf},\;V_{e},T_{tp},{\bar{P}}_{nf},C_{nf},\;P_{tp}\right\}$$

Definitions for the correlated variables of thermoelectric values include the thermal conductivity of the novel TEP, $K_{tp}$, the thermal conductivity of nanofluid, $K_{nf}$, the nanofluid specific heat, $C_{nf}$, the nanofluid viscosity, $\mu_{nf}$, the nanofluid density, $\rho_{nf}$, the temperature of novel TEP, $T_{tp}$, the filling amount of novel TEP, $F_{tp}$, the pressure of novel TEP, $P_{tp}$, the zeta potential of nanofluid, $V_{n}$, the electric charge density output of the novel TEP with nanofluid as electrolyte, ${\bar{P}}_{tp}$, the standard electric potential of the novel TEP electrode, $V_{e}$, and the electric charge density output of the copper-aluminum battery cell with nanofluid as electrolyte, ${\bar{P}}_{nf}$. We determined the relevant physical quantities and expressed the variables as the basic physical quantities, then selected the required variables to represent each Π term and repeated variables. We then calculated through repeated variables and multiplied each Π term to find the dimensionless parameters of each Π term. The obtained Π term is expressed as a functional relationship. Equation (1) reveals the $K_{tp}$ function, which was defined via the other seven variables, four of which were independent physical quantities, namely, mass (M), length (L), time (T), and temperature (Θ). ${\bar{P}}_{tp}$ was decided by the other nine variables, five of which were independent physical quantities consisting of M, L, T, Θ, and voltage (V). These can be employed as in Equation (2). Expressions of all variables were adopted through the M, L, T, Θ, and V, as follows: they are $\;K_{tp}=\;\left[{\mathrm{MLT}}^{-3}\theta^{-1}\right]$, $K_{nf}=\;\left[{\mathrm{MLT}}^{-3}\theta^{-1}\right]$, $C_{nf}=\;\left[L^{2}T^{-2}\theta^{-1}\right]$, $\mu_{nf}=\;\left[{\;\mathrm{ML}}^{-1}T^{-1}\right]$, $\rho_{nf}=\;\left[{\mathrm{ML}}^{-3}\right]$, ${\bar{P}}_{nf}=\;\left[{\mathrm{MT}}^{-3}\right]$, $T_{tp}=\;\left[\theta \right]$, $F_{tp}=\;\left[L^{3}\right]$, $P_{tp}=\;\left[{\;\mathrm{ML}}^{-1}T^{-2}\right]$, $V_{n}=\;\left[V\right]$, and ${\bar{P}}_{tp}=\;\left[{\mathrm{MT}}^{-3}\right]$. Regarding the effective thermal conductivity of the novel TEP, there are four dimensionless Π numbers for the $K_{tp}$. This study chose four repeating variables, $K_{nf}$, $\rho_{nf}$, $F_{tp}$, and $C_{nf}$ for extrapolations. These four repeating variables are multiplied through other non-repeating variables to gain the dimensionless Π parameters. The four Π groups are shown in Equation (3). For the electric charge density of the novel TEP, ${\bar{P}}_{tp}$, five dimensionless Π number groups are determined separately and five repeated variables (${\bar{P}}_{nf}$, $V_{e}$, $F_{tp}$, $\rho_{nf}$, and $C_{nf}$) are chosen. The analysis procedure is the same as the empirical formula of Equation (3). Equation (2) displayed the electric charge density output functional equation. The five Π groups are exhibited in Equation (4). The known attributes of the novel TEP acquired from the thermoelectric experiment and experimental data are substituted into Equations (3) and (4) to obtain the indeterminate values of α, β, γ, λ, and τ. After simplification, the thermoelectric empirical formula of the novel TEP are derived in the present study.

(3) $$\frac{K_{tp}}{{\;K}_{\mathrm{nf}}}=\alpha {\left\{\left.\frac{\mu_{\mathrm{nf}}\cdot \;F_{\mathrm{tp}}{}^{\frac{4}{3}}\cdot \;C_{\mathrm{nf}}}{K_{\mathrm{nf}}}\right\}\right.}^{\beta }{\left\{\left.\frac{T_{tp}\cdot \;\rho_{\mathrm{nf}}{}^{2}\cdot \;F_{\mathrm{tp}}{}^{\frac{2}{3}}\cdot \;C_{\mathrm{nf}}{}^{3}}{K_{\mathrm{nf}}{}^{2}}\right\}\right.}^{\gamma }{\left\{\left.\frac{P_{\mathrm{tp}}\cdot \;\rho_{\mathrm{nf}}\cdot \;F_{\mathrm{tp}}{}^{\frac{2}{3}}\cdot \;C_{\mathrm{nf}}{}^{2}}{K_{\mathrm{nf}}{}^{2}}\right\}\right.}^{\lambda }$$

(4) $$\frac{{\bar{\text{P}}}_{\mathrm{tp}}}{{\bar{\text{P}}}_{\mathrm{nf}}}=\alpha {\left\{\frac{V_{n}}{V_{t}}\right\}}^{\beta }{\left\{\frac{\mu_{\mathrm{nf}}}{{\bar{\text{P}}}_{\mathrm{nf}}{}^{\frac{1}{3}}\cdot \;F_{\mathrm{tp}}{}^{\frac{1}{3}}\cdot \;\rho_{\mathrm{nf}}{}^{\frac{2}{3}}}\right\}}^{\gamma }{\left\{\frac{T_{\mathrm{tp}}\cdot \;\rho_{\mathrm{nf}}{}^{\frac{2}{3}}\cdot \;C_{\mathrm{nf}}}{{\bar{\text{P}}}_{\mathrm{nf}}{}^{\frac{2}{3}}}\right\}}^{\lambda }{\left\{\frac{P_{\mathrm{tp}}\cdot \;F_{\mathrm{tp}}{}^{\frac{1}{3}}}{{\bar{\text{P}}}_{\mathrm{nf}}{}^{\frac{2}{3}}\cdot \;\rho_{\mathrm{nf}}{}^{\frac{1}{3}}}\right\}}^{\tau }$$

This research reports the intelligent dimensional analysis methods [22] to be used to find the effective thermal conductivity values and the electric charge density output values of the novel TEP. Consequently, inevitable errors certainly exist among the real values owing to the artificial operation, the restriction of preciseness of the experimental instrument, the measured data during experiment, and the values deriving from experimental data. It is essential to premeditate the trial errors so as to find the experimental reliance before resolving the experimental results based on this, where the notion of the error propagation is employed to appraise the experimental errors and basic functionary relations. Many items of effective thermal conductivities and electric charge density outputs are applied separately to survey the effective thermal conductivity values and the electric charge density output values of the novel TEP during the thermoelectric experiments. The effective thermal conductivity values and electric charge density output values respectively pertain to derived variables consisting of $K_{tp}$, $K_{nf}$, $C_{nf}$, $\mu_{nf}$, $\rho_{nf}$, $T_{tp}$, $F_{tp}$, $P_{tp}$, $V_{n}$, ${\bar{P}}_{tp}$, $V_{e}$, and ${\bar{P}}_{nf}$ which are measured with experimental apparatus. The error of experimental apparatus is propagated to the consequence during deduction, and thereby transforms the errors of effective thermal conductivities and electric charge density output values. The experimental error is indicated with a relative error, and the maximum relative errors of effective thermal conductivities and electric charge density outputs are within ±5.5% and ±10%, respectively. The experimental results are exploited in dimensional analysis to derive the empirical formulas of the novel TEP.

### 2.2. Structural Design of the Novel TEP and Experimental Apparatus

**Figure 1:**
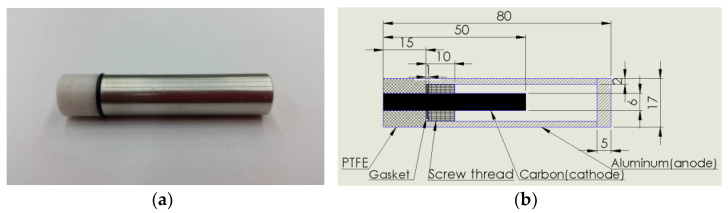
The Novel TEP device. (**a**) Entity photo, (**b**) dimensions and materials.

Figure 1 demonstrates that the novel TEP adopts materials including polytetrafluoroethylene (PTFE), Viton rubber (Gasket), carbon, and aluminum. The carbon rod and the aluminum tube with good thermal conductivities are respectively used as the cathode and the anode. In the insight design, the aluminum and carbon are insulated with PTFE and gaskets to avoid short circuits. Afterwards, the ionic compounds and the nanofluids possessing good thermal conductivity were filled into the tube as the electrolytes, which had four types, involving titanium oxide nanofluid, deionized water, surface seawater, and polymeric nanofluids composed of nanoparticles added to sodium hydroxide (NaOH). When the tubular electrode (aluminum tube) and the core rod (carbon rod) electrode have a temperature difference, thermal energy can be directly converted into electric energy by the redox reaction of the electrolytes (four kinds), and the electrodes can generate electromotive force. In particular, the novel PET device may use the structural design between the tubular electrode and the core rod electrode to provide a greater contact area with a heat source, and may be directly immersed in a heat source.

The principal design of the TEP is based on the energy conservation theorem. Thermal energy increased the rate of the redox reaction, which affected the current density and transferred the energy through electrons to generate electrical energy. The novel TEP is currently devised as a cylinder with a diameter of 17 mm and a length of 80 mm, as shown in the Figure 1b. The operating principle is that the heat energy generated by the machine tool or heat source was transferred to the novel TEP and then the temperature of the nanofluid inside the tube was raised in order to increase the redox reaction rate of the novel TEP, thereby generating additional output power and transferring the power to the carrier for heat recycling.

**Figure 2:**
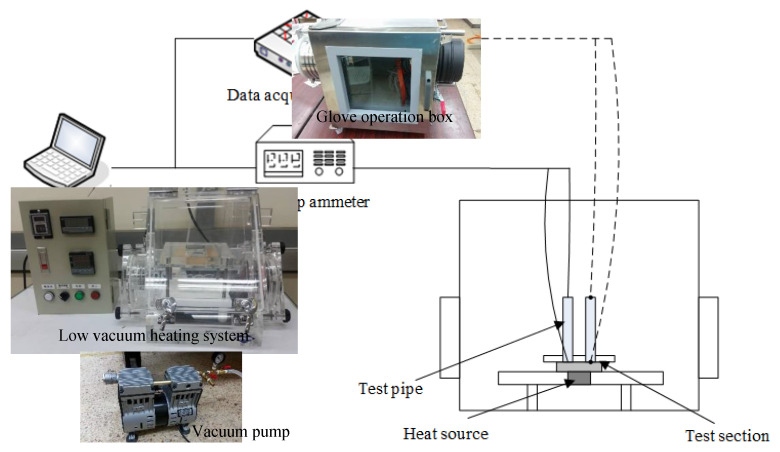
Thermoelectric performance experiment framework of the TEP.

The experimental framework of the present study is shown in Figure 2. The low-vacuum heating system used in the experiment was manufactured by the thermal-fluid illumination laboratory of National Taiwan Ocean University (NTOU) from Keelung in Taiwan, for which the operating temperature was between 25 and 120 °C and the operating pressure was 300 to 760 torr. The novel TEP was fixed on the heating platform and the operation control panel regulated the time and heating temperature. The low-vacuum glove operation box was employed to fill the electrolytes into the novel TEP, which was from Hoyu Technology Co., Taipei, Taiwan. The oil-free vacuum pump was utilized for low vacuum pressure with a voltage of 100 to 115 V, a motor power of 560 W, and an exhaust speed of 100 l/min. Experimental temperatures were measured by the T-type copper-nickel thermocouples with a wire diameter of 0.32 mm, occupying a measuring range between –200 and 350 °C and an error range of ±0.5 °C. The data logger of the GL-800 was made by Graphtec Co., Yokohama, Japan, which had 40 measuring items containing temperature, voltage, and humidity, etc., with a sampling time of 0.1 ms and a measurement error of ±1%. In the present experiment, the temperature data of the thermocouple can be captured and recorded on the hard disk and output as a Microsoft Office Excel table. The digital electric meter of TM-8155 with the measurement accuracy of ± (0.05% reading + 5 digits) used in the experiment was produced by Twintex Instrument Co., New Taipei City, Taiwan, which had the functions of measuring voltage, current, resistance, capacitance, and so on. The generated power density of the novel TEP was determined by the TM-8155. Eventually, the maximum measuring error for the thermoelectric performances of the novel TEP device was thus within ± 3%.

### 2.3. Thermoelectric Function Testing of the Novel TEP

**Figure 3:**
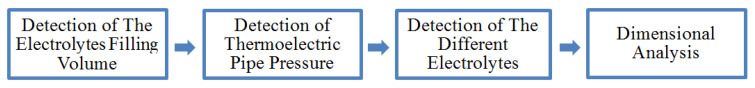
Thermoelectric function testing process of the novel TEP.

The performance test of the novel TEP was mainly divided into two parts. The first was to discuss the thermoelectric performance test of the novel TEP using the titanium dioxide nanofluid as the electrolyte under different filling amounts and pressures. Afterwards, the thermoelectric function test of four different electrolytes, including nanofluid, seawater, deionized water, and polymeric nanofluids, composed of nanoparticles added to sodium hydroxide filled into the novel TEP at different temperatures, was investigated. The experimental process of thermoelectric performance is shown in Figure 3.

**Figure 4:**
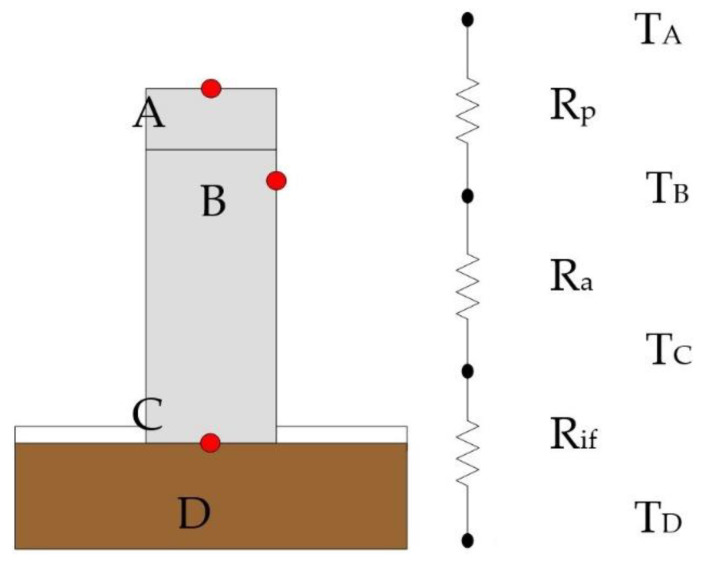
Thermal resistance network of the novel TEP.

The present research mainly exploits the one-dimensional thermal resistance model [22] to evaluate the heat dissipation characteristics of the novel TEP, and the thermal resistance was used to judge the heat dissipation capacity. The larger the thermal resistance value was, the worse the heat dissipation effect was. The thermal resistance of the interface between the heat source and TEP was controlled between 0.05 and 0.09 °C/W. In addition, the thermal conductivity of the novel TEP was derived through the one-dimensional Fourier heat conduction equation. Thermoelectric performance experiments were carried out in different filling ratios of the novel TEP electrolyte, tube pressures, and electrolyte compositions, and thermocouples were employed to detect the heat transfer of the novel TEP under different temperatures. The temperature difference between the bottom and the top of the novel TEP was adopted to calculate the thermal resistance of the TEP, and then the thermal conductivity of the novel TEP was derived. The thermocouple measurement temperature points and thermal resistance analysis are shown in Figure 4. T_A_ is the top surface temperature of TEP, T_B_ is the side surface temperature of TEP, T_C_ is the bottom surface temperature of TEP, and T_D_ is the heating source temperature.

The effects of filling ratio and vacuum pressure for the novel TEP are major parameters on the thermal performance. In the low-vacuum glove operation box, the oil-free pump was used to evacuate the pressure to the experimental test. The electrical property experiments of the novel TEP with four different solutions involving seawater, deionized water, nanofluid, and polymeric nanofluid are the output current density and power density between 25 and 100 °C. The filling electrolyte processes of the novel TEP are as follows: firstly, fix the TEP in the low-vacuum glove operation box and open the tube cover. When the vacuum pressure reaches the experimental pressure via vacuum pump, close the suction valve. Use the operating gloves to tighten the tube and the cap of the novel TEP. Open the suction valve to make the box pressure reach normal pressure. Then, fix the TEP on the heating platform of the low-vacuum heating system with a downward force of 14.7 N through the baffle plate. The filling ratio experiment was inspected at 400 torr vacuum pressure, at 7 mL (100%), 5.6 mL (80 %), 4.2 mL (60%), 2.8 mL (40%), and 0 mL (0%), and the vacuum pressure tests were 400, 500, 600, and 760 torr, respectively. The experimental processes of the thermoelectric performance were as follows. First, fix the TEP tube on the heating platform, and apply heat dissipation paste on the bottom to make the heat transfer uniform. Connect to the anode of the aluminum tube and cathode of the carbon rod respectively, and connect a desktop electric meter. Power on the operating platform and set the heat source temperature, and start the heating device and set the heating time to 40 min. When the temperature, current, and voltage of the heat source stabilize and reach the experimental temperature, record the temperatures, output current, and voltage. Finally, adjust the heat source to the experimental temperature of 25 to 100 °C, and reset the heating time. Repeat the above the steps and measure temperature, current, and voltage of the novel TEP for different vacuum pressures and filling ratios under four different electrolytes.

## 3. Results

The thermoelectric performances of the novel TEP under different conditions involving the temperatures, pressures, and better nanofluids as electrolytes are investigated in the present work. Through intelligent experiments, obtained data can be utilized to summarize the empirical formulas of the novel TEP thermoelectric performances by the dimensional analysis method [22].

### 3.1. Effect of Adding Ionic Compounds on Nanofluids

**Figure 5:**
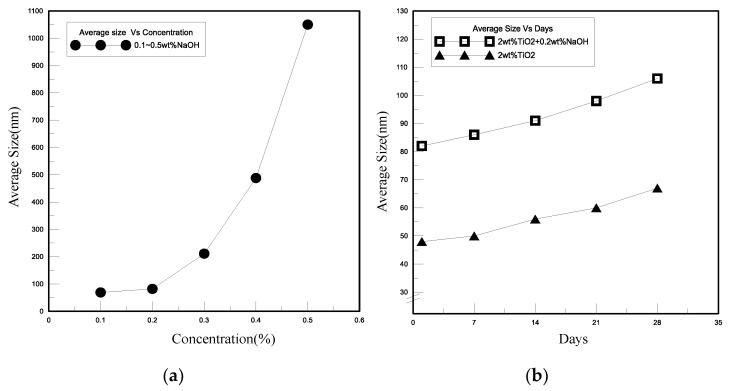
The effect of sodium hydroxide (NaOH) on titanium dioxide (TiO_2_) nanofluid. (**a**) Different concentrations of NaOH, (**b**) particle size.

To explore the effect of adding different concentrations of sodium hydroxide (NaOH) on the particle size and suspension of the polymeric nanofluids, this study appended a better concentration of NaOH to the 2 wt.% titanium dioxide (TiO_2_) nanofluid to improve the electrical properties of the novel TEP device. NaOH is an ionic compound that will completely dissociate into sodium ions (Na^+^) and hydroxide ions (OH^-^) in an aqueous solution, which increases the number of ions that react with the cathode of aluminum in the solution to raise the amount of current. Figure 5a exhibits the effect of adding 0.1 to 0.5 wt.% NaOH on the particle size of nanofluid in 2 wt.% TiO_2_ nanofluid. The average size of nanoparticle of polymeric nanofluids was less than 100 nm when adding lower concentrations of 0.1 to 0.2 wt.% NaOH. When 0.3 wt.% NaOH was appended, the particle size was 211 nm, and the particle sizes were 488 and 1050 nm respectively, when 0.4 to 0.5 wt.% NaOH was added. NaOH will erode the surface of nanoparticles inside the metallic nanofluids as it reaches a certain concentration, resulting in an agglomeration phenomenon. Therefore, for the stability and suspension of polymeric nanofluids, we chose to add a lower concentration of NaOH to reduce the impact on nanoparticles. In Figure 5b, comparing the effect of adding 0.2 wt.% NaOH on the particle size, the overall increase in the particle size of the TiO_2_ nanofluid with 0.2 wt.% NaOH was about 39%. Adding ionic compounds into the titanium dioxide nanofluids in this experiment, 0.2 wt.% NaOH has tiny effects on the stability of nanoparticles and suspension by detecting particle size and concentration of the polymeric nanofluids, resulting in improving the electrical properties of the present novel TEP.

### 3.2. Effect of Filling Quantity on Thermoelectric Performance of the Novel TEP

**Figure 6:**
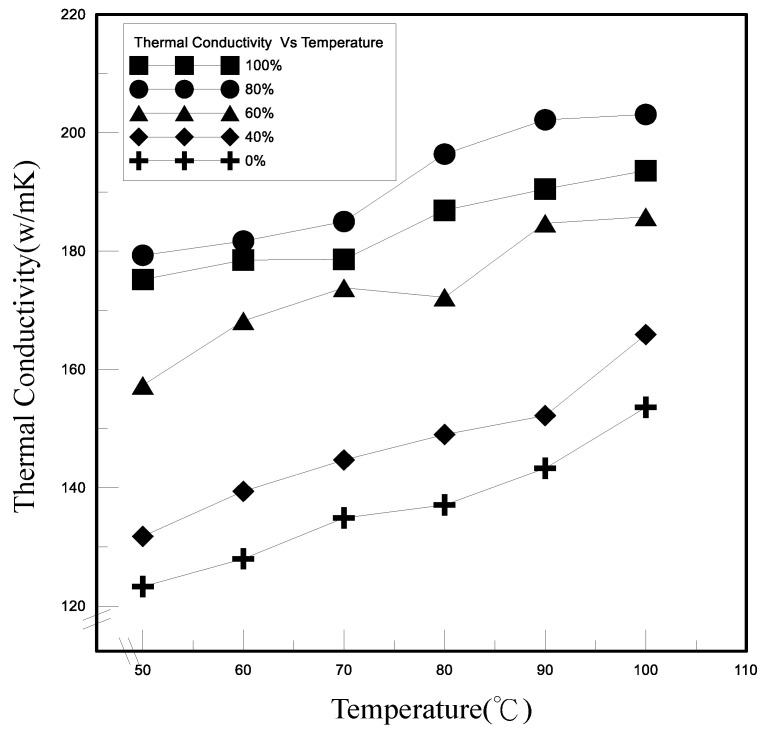
Relationship between thermal conductivity and temperature of TEP.

**Figure 7:**
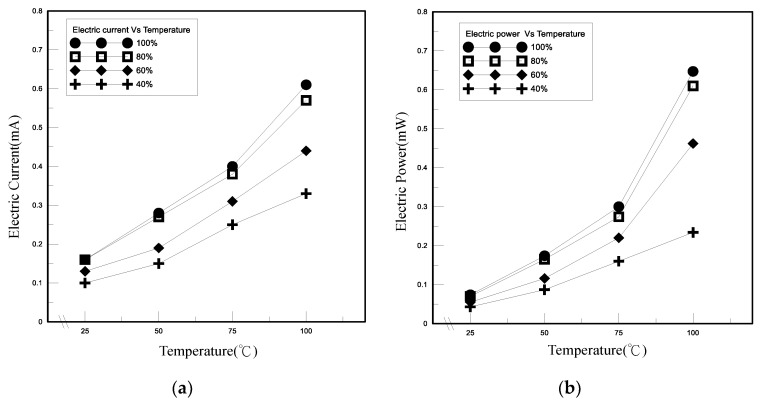
Electric performances of TEP under different filling ratios. (**a**) Current, (**b**) power.

As shown in Figure 6, the heat transfer coefficients of the novel TEP tended to increase as the polymeric nanofluid filling amount increased under the pressure of 400 torr in the tube. The thermal conductivity of the novel TEP is derived through the one-dimensional Fourier heat conduction equation and one-dimensional thermal resistance model. We controlled the experimental temperature of the heat source between 25 and 100 °C and let it operate at heating time until steady state. Between 50 and 60 °C, for the filling ratio in the tube of 100% and 80%, the difference in the thermal conductivity was a small increase of about 2%. However, the difference in the coefficient of thermal conductivity of the filling ratio of 100% and 80% in the tube is about 5% above 80 °C. Consequently, the phase change of the fluid increases as the temperature rises. At high temperature, the novel TEP with 80% filling ratio has more space for phase change and takes away more thermal energy, resulting in high thermal conductivity. The current and power of the novel TEP tended to increase as the temperature and filling ratio increased, as shown in Figure 7. When the solution was heated, the evaporation rate of liquid increased to take away more thermal energy, resulting in increasing the thermal conductivity of the novel TEP. Nevertheless, the distinction of the current and power of the novel TEP between 100% and 80% filling ratios was slight because of the novel TEP structural design factors, in which the difference in the electrode reaction area contacted by the filling ratios of 80% and 100% was not large. The electrode reaction surface area of the filling ratio of 100% was only 3.2 cm^2^ larger than that of the filling ratio of 80%. The positive reaction was conducive to gas generation. The higher amount of space is conducive to the generation of redox reaction gas of the novel TEP for the filling ratio of 80%, so that the temperature of the polymeric nanofluid in the tube is higher and the electrical energy is increased.

### 3.3. Effect of Vacuum Pressure on Thermoelectric Performance of the Novel TEP

Table 1 displayed that the thermal conductivities of the novel TEP tended to rise as the vacuum pressure decreased, resulting from the increase in phase change phenomena of the polymeric nanofluid. The overall increment of the thermal conductivity coefficient was about 10% under the vacuum pressure in the TEP tube decreasing from 760 to 400 torr. The relationships between electric performances and temperatures of the novel TEP at different vacuum pressures are shown in Table 2. The power density increased while reducing the vacuum pressure and increasing the thermal conductivity during the heating processes, and the overall temperature of the internal fluid increased, resulting in increasing the power density. These electric performances were current density and power density based on the electrode reaction surface area of the novel TEP, of 24.76 cm^2^. Table 2 exhibited that the current and power densities were not greatly affected by vacuum pressure at 25 °C. However, with the increase in temperature and the decrease in vacuum pressure of the novel TEP, the current and power densities had an increasing trend. As the vacuum pressure decreased, the boiling point of the solution inside the tube decreased and the power density tended to increase. Since the reduced vacuum pressure was conducive to the formation of positive electrochemical reactions and the reduction of the thermal resistance inside the tube, the temperature of the polymeric nanofluid inside the tube increased, so that the overall current and power densities rose. In addition, more ions were transported to the electrodes through the auxiliary driving forces of the chemical gradient and potential activity. Reducing the vacuum pressure of the tube increased the rate of the redox reaction, thereby improving the electrical performance. Finally, the overall growth rate was respectively about 22% and 28%.

### 3.4. Effect of Different Electrolytes on Thermoelectric Performance of the Novel TEP

**Figure 8:**
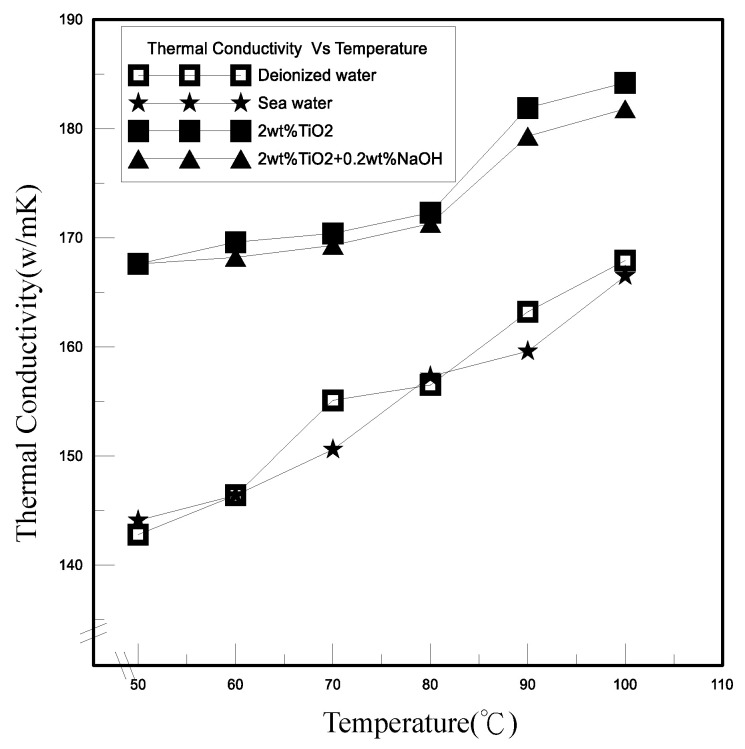
Relationship between thermal conductivity and temperature of different electrolytes of the novel TEP.

**Figure 9:**
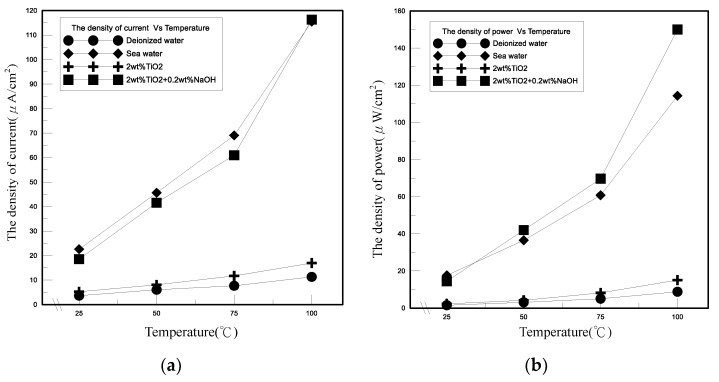
Relationship between electric performances and temperatures of different electrolytes of the novel TEP. (**a**) Current, (**b**) power.

Figure 8 demonstrates the thermal conductivities with temperatures change of the novel TEP under various electrolytes, including the deionized water, seawater, titanium dioxide nanofluid, and polymeric nanofluid composed of titanium dioxide nanoparticles with sodium hydroxide. The thermal conductivity of TiO_2_ nanofluid was the best among these four solutions. The polymeric nanofluid composed of TiO_2_ nanoparticles added to NaOH affects the nanoparticle size, resulting in more serious agglomeration between the nanoparticles and the lower thermal conductivity of NaOH, so that its thermal conductivity tended to decrease more than TiO_2_ nanofluid. The difference of thermal conductivity coefficient between seawater and deionized water was not much different and their thermal conductivity coefficients were relatively poor. Compared with deionized water, the overall increase of thermal conductivity coefficient of the novel TEP containing TiO_2_ nanofluid was about 11%. The relationships between electric performances and temperatures of the novel TEP at different electrolytes are shown in the Figure 9. However, the effects of different electrolytes on the novel TEP could be seen in that the electric performances of the polymeric nanofluid was the highest among them, and the difference between seawater and the polymeric nanofluid was not much different. Compared with deionized water and TiO_2_ nanofluid from Figure 9a, the current density was relatively low. The overall increases in current density of TiO_2_ nanofluid and the polymeric nanofluid were respectively about 45% and 419%. Compared with deionized water and TiO_2_ nanofluid from Figure 9b, the power density was relatively low. The overall increases in power density of TiO_2_ nanofluid and the polymeric nanofluid were respectively about 55% and 768%.

### 3.5. Thermal Conductivity and Power Density Empirical Formulas of the Novel TEP

In the present study, the purpose of deducing these empirical formulas, including thermal conductivity and power density of the TEP, is to utilize the intelligent dimensional analysis [22] to obtain these values under certain conditions and to enter several basic parameters that did not require instrument measurement. Comprehensive parameters in the empirical formulas were used to forecast the thermoelectric performances of the TEP in the present paper. According to the empirical formulas of the novel TEP derived from the experimental data, they were applicable for 2 wt.% titanium dioxide nanofluids between 25 and 100 °C with vacuum pressure between 400 and 760 torr and with an 80% filling ratio.

Originally, for the thermal conductivity formula of the novel TEP, we substituted the experimental parameters of $K_{nf}$, $\rho_{nf}$, $F_{tp}$, $C_{nf}$, and $\mu_{nf}$ into Equation (3). In order to facilitate a simple calculation, smaller changes of $\rho_{nf}$, $C_{nf}$, and $\mu_{nf}$ will be substituted into Equation (3) by mean values at different temperatures. As the product of $\left(C_{nf}\;\cdot \;\mu_{nf}\right)$ was a negligible change as vacuum pressure increased, it was ignored because of no impact on the thermal conductivity of TEP and then it was assumed that  β = 0. Therefore, Equation (5) can be briefly acquired as:

(5) $$K_{tp}=0.62\alpha {\left(50270.55T_{tp}\right)}^{\gamma }{\left(13.17P_{tp}\right)}^{\lambda }$$

Equation (5) illustrated that $K_{tp}$ was determined by the temperature and vacuum pressure of the novel TEP. The factor of vacuum pressure was temporarily not pondered. Setting λ = 0 and taking 400 torr as a benchmark revealed that $K_{tp}$ changed along with temperature at 400 torr. Substituting values from Table 3 into Formula (5), we obtained Equation (6):

(6) $$K_{tp}=1.2042T_{tp}{}^{0.866}$$

The parameters in Table 3 displayed that the changes in temperature and thermal conductivity of TEP are similar at different vacuum pressures. Consequently, λ value and polynomial function were estimated through the parameters of Table 3 based on 50 °C as the basis under different vacuum pressures. The vacuum pressure was then a function of λ. In summary, the final empirical formula of the novel TEP thermal conductivity is Equation (7):

(7) $$K_{tp}=1.2042T_{tp}{}^{0.866}{\left(13.17P_{tp}\right)}^{\lambda }\;\lambda =-5.10769\times 10^{-10}P_{tp}{}^{3}+9.69923\times 10^{-7}P_{tp}{}^{2}-6.1349\times 10^{-4}P_{tp}+0.1229$$

For the power density formula of the novel TEP, the known parameters were substituted into Formula (4). Since the dimensionless parameters were regarded as having little effect on the power density of the novel TEP, the index was assumed to be zero, the same as the processes of deriving the empirical formula of TEP thermal conductivity. Subsequently, the derived results were exhibited in Equation (8), resulting from Equation (4):

(8) $${\bar{\text{P}}}_{\mathrm{tp}}=0.34\alpha {\left(209.19T_{tp}\right)}^{\lambda }{\left(0.084P_{tp}\right)}^{\tau }$$

Without considering the factor of vacuum pressure first, τ was assumed to be zero when the vacuum pressure of 400 torr was utilized as a benchmark to find the functional relationship of the power density and temperature at this pressure, as shown in Equation (9):

(9) $${\bar{\text{P}}}_{\mathrm{tp}}=\;\left(2.167\times 10^{-23}\right)T_{tp}{}^{9.333}$$

Finally, vacuum pressure was taken into consideration and 50 °C was employed as the benchmark to find the index for τ value. Equation (10) illustrates the power density empirical formula of the novel TEP:

(10) $${\bar{\text{P}}}_{\mathrm{tp}}=\;\left(2.167\times 10^{-23}\right)T_{tp}{}^{9.333}{\left(0.084P_{tp}\right)}^{\tau }\;\tau =9.1203\times 10^{-9}P_{tp}{}^{3}-1.5595\times 10^{-5}P_{tp}{}^{2}+8.4313\times 10^{-3}P_{tp}-1.461$$

As mentioned above, many problems still exist in the present thermoelectric experiments that cause error rates for thermal conductivity and low and unstable current and power output. The calculation method of the error rate is shown in Formulae (11) and (12), where E_K_ is the error value between the instrument value of K_tp,e_ measured by the equipment and the calculated value of K_tp,f_ calculated by the empirical formula of the thermal conductivity coefficient of the novel TEP. E_P_ is the error value between the instrument value of ${\bar{P}}_{tp,e}$ measured by the equipment and the calculated value of ${\bar{P}}_{tp,f}$ calculated by the empirical formula of the power density of the novel TEP. Table 4 and Table 5 illustrate the error rates of E_K_ and E_P_. Initial estimations show that the calculated and measured values were similar. The derived empirical formulas were calculated based on the vacuum pressure of 400 torr and temperature of 50 °C, in which the error rates of the calculated value and the measured value will be lower, and the largest error rate of the thermal conductivity coefficient was 5.06%, indicating that the overall error rates were small. The power density will affect the stabilities of the current and voltage due to the oxidation of the aluminum electrode, and the longer the reaction time, the more the current will decay, and thus the error rate will also be affected. The maximum error rate of power density was 11.27%.

(11) $$E_{K}=\frac{K_{tp,e}-K_{tp,f}}{K_{tp,e}}\times 100\%$$

(12) $$E_{p}=\frac{{\bar{P}}_{tp,e}-{\bar{P}}_{tp,f}}{{\bar{P}}_{tp,e}}\times 100\%$$

## 4. Conclusions

The empirical formulae including both thermal conductivity and power density for the TEP of TiO_2_ nanofluid adopted the dimensional analysis method with thermoelectric experimentation and were applicable to temperatures between 25 and 100 °C and vacuum pressures between 400 and 760 torr in this paper, which were verified and not ordinary expressions. Their error values were separately less than 5.1% and 11.3%. The present empirical equations were not used to forecast the exact values, but instead of portending the thermodynamic activities of the novel TEP under various temperatures and vacuum pressures. For the thermal and electrical performance experiments with different filling volumes, the performances of the novel TEP tended to increase as the filling amount increased. The vacuum pressure inside the tube also affected the electrical and thermal performances of the novel TEP. The results indicated that the novel TEP with a filling ratio of 80% had better performance resulting from the space inside the tube required for the liquid–gas phase conversion to improve heat dissipation and power generation performances. The thermal performance of titanium dioxide nanofluid of the novel TEP and the electrical performance of the polymeric nanofluid of the novel TEP were respectively better among TiO_2_ nanofluid, TiO_2_ nanofluid with 0.2 wt.% NaOH, deionized water, and seawater. The temperature and vacuum pressure gradients of chemical and thermodynamic potentials were the driving forces, which had a significant role in the thermoelectric experiment. Finally, the formulas were suitable for an approximate estimation in the present study.

## Figures and Tables

**Table 1:** Thermal conductivities W/(m·K) of the novel TEP under various pressures.

	Pressure (torr)	760	600	500	400
Temp. (°C)					
TEP	50	167.6	169.4	171.3	179.3
	60	169.6	171.0	177.0	181.7
	70	170.4	172.7	178.6	185.0
	80	172.3	179.3	185.8	196.4
	90	181.9	184.7	188.5	202.2
	100	184.2	186.7	190.1	203.1

**Table 2:** Electric performances (µA/cm^2^ and µW/cm^2^) of the novel TEP under various pressures.

	Pressure (torr)	760	600	500	400
Temp. (°C)					
Current density (µA/cm^2^) of TEP	25	5.25	5.25	5.65	5.65
	50	8.08	8.89	10.10	10.10
	75	11.71	13.33	14.54	14.94
	100	16.96	18.58	21.01	21.81
Power density (µW/cm^2^) of TEP	25	2.30	2.30	2.59	2.67
	50	4.20	4.44	5.25	5.33
	75	8.20	9.21	10.34	10.74
	100	15.11	16.72	19.55	21.16

**Table 3:** Parameters substituted into the empirical formulas of the novel TEP.

C_nf_ (J/g-K)			1.45			
ρ_nf_ (kg/m^3^)			1084.16			
μ_nf_ (cP)			1.45			
F_tp_ (cm^3^)			5.60			
V_t_ (V)			1.79			
A (cm^2^)			24.76			
K_nf_ (W/m-K)			0.62			
${P\text{¯}}_{\mathrm{nf}}$ (μW/cm^2^)			2.95			
V_n_ (mV)			0.47			
${\bar{\text{P}}}_{\mathrm{tp}}$ (μW/cm^2^)		Pressure (torr)	760	600	500	400
	Temp. (°C)					
	25 (298 K)		2.30	2.30	2.59	2.67
	50 (323 K)		4.20	4.44	5.25	5.33
	75 (348 K)		8.20	9.21	10.34	10.74
	100 (373 K)		15.44	16.72	19.55	21.16
K_tp_ (W/m-K)	50 (323 K)		167.6	169.4	171.3	179.3
	60 (333 K)		169.6	171.0	177.0	181.7
	70 (343 K)		170.4	172.7	178.6	185.0
	80 (353 K)		172.3	179.3	185.8	196.4
	90 (363 K)		181.9	184.7	188.5	202.2
	100 (373 K)		184.2	186.7	190.1	203.1

**Table 4:** Comparison of measured and calculated thermal conductivities W/(m·K) of TEP and error rates under different vacuum pressures.

Temperature (°C)		50	60	70	80	90	100
760 torr	Equipment	167.60	169.60	170.40	172.30	181.90	184.20
	Formula	167.61	172.10	176.56	181.01	185.44	189.86
	E_K_ (%)	–0.01	–1.47	–3.62	–5.06	–1.95	–3.07
600 torr	Equipment	169.40	171.00	172.70	179.30	184.70	186.70
	Formula	169.40	173.94	178.45	182.95	187.43	191.89
	E_K_ (%)	0	–1.72	–3.33	–2.04	–1.48	–2.78
500 torr	Equipment	171.3	177.00	178.60	185.80	188.50	190.10
	Formula	171.3	175.89	180.45	185.00	189.53	194.04
	E_K_ (%)	–0.01	0.63	–1.04	0.43	–0.55	–2.07
400 torr	Equipment	179.30	181.70	185.00	196.40	202.20	203.10
	Formula	179.33	184.13	188.91	193.67	198.42	203.14
	E_K_ (%)	–0.02	–1.34	–2.11	1.39	1.87	–0.02

**Table 5:** Comparison of measured and calculated power densities μW/cm^2^ of TEP and error rates under different vacuum pressures.

Temperature (°C)		25	50	75	100
760 torr	Equipment	2.30	4.20	8.20	15.44
	Formula	2.11	4.48	8.97	17.15
	E_p_ (%)	8.34	–6.56	–9.46	–11.27
600 torr	Equipment	2.30	4.44	9.21	16.72
	Formula	2.23	4.73	9.49	18.14
	E_p_ (%)	3.05	–6.54	–3.09	–8.47
500 torr	Equipment	2.59	5.25	10.34	19.55
	Formula	2.64	5.59	11.22	21.43
	E_p_ (%)	–2.02	–6.54	–8.48	–9.63
400 torr	Equipment	2.67	5.33	10.74	21.16
	Formula	2.68	5.68	11.39	21.76
	E_p_ (%)	–0.43	–6.52	–5.97	–2.81

## Data Availability

All data are offered by the authors for reasonable request and the novel TEP device is available from the authors.
